# Alcohol use and the gender-specific risk of suicidal behavior: a systematic review and meta-analysis protocol

**DOI:** 10.1186/s13643-022-02159-0

**Published:** 2022-12-23

**Authors:** Shannon Lange, Michael Roerecke, Heather Orpana, Courtney Bagge, Jürgen Rehm

**Affiliations:** 1 Institute for Mental Health Policy Research, Centre for Addiction and Mental Health, 33 Ursula Franklin St. T521, ON M5S 2S1 Toronto, Canada; 2 Campbell Family Mental Health Research Institute, Centre for Addiction and Mental Health, 250 College St., Toronto, ON M5T 1R8 Canada; 3 Department of Psychiatry, University of Toronto, 250 College St., Toronto, ON M5T 1R8 Canada; 4 Dalla Lana School of Public Health, University of Toronto, 155 College St, Toronto, ON M5T 3M7 Canada; 5 Public Health Agency of Canada, 785 Carling Ave, Ottawa, ON K1A 0K9 Canada; 6 School of Epidemiology and Public Health, University of Ottawa, 600 Peter Morand Cres, Ottawa, ON K1G 5Z3 Canada; 7 Royal Ottawa Institute for Mental Health Research, 1145 Carling Ave, Ottawa, ON K1Z 7K4 Canada; 8 Department of Psychiatry, University of Michigan Medical School, 1500 E Medical Center Dr, Ann Arbor, MI 48109 USA; 9 Department of Veterans Affairs, Center for Clinical Management Research, 2215 Fuller Rd., Ann Arbor, MI 48105 USA; 10 Institute of Medical Science, University of Toronto, 1 King’s College Circle, Toronto, ON M5S 1A8 Canada; 11 Institute of Clinical Psychology and Psychotherapy, Technische Universität Dresden, Chemnitzer Str. 46, 01187 Dresden, Germany; 12 Department of International Health Projects, Institute for Leadership and Health Management, I.M. Sechenov First Moscow State Medical University, Trubetskaya Street 8, B. 2, Moscow, 119991 Russian Federation; 13 Zentrum Für Interdisziplinäre Suchtforschung (ZIS), Universitätsklinikum Hamburg-Eppendorf, Martinistraße 52, 20246 Hamburg, Germany

**Keywords:** Alcohol use, Death by suicide, Gender, Suicidal behavior, Suicide attempt, Risk relationship

## Abstract

**Background:**

Alcohol use is an important risk factor for suicidal behavior, with a heightened risk found among women. The objective of this study is to determine the gender-specific risk of suicidal behaviors (suicide attempt and death by suicide) for different levels and dimensions of alcohol use—i.e., for (1) average alcohol volume consumed, (2) binge drinking, and (3) individuals with an alcohol use disorder.

**Methods:**

We will systematically search the available literature for primary studies on the risk relationships specified above. Using a predetermined set of keywords, a comprehensive systematic literature search will be conducted in the following electronic databases: Embase, PsycINFO, PubMed, and Web of Science. The basic inclusion criteria will be (1) an original, quantitative (cohort, case–control or cross-sectional) study; with (2) a measure of risk of at least one dimension of our alcohol exposures in relation to at least one of our outcomes of interest (suicide attempt or death by suicide), and its corresponding measure of variability is reported (or sufficient data to calculate these); and (3) estimates of risk stratified by gender. Studies (1) that use only qualitative labels of alcohol use, and (2) where suicide attempt and non-suicidal self-harm cannot be disaggregated will be excluded. There will be no restrictions on language, geographical region, or year of publication. Two reviewers will independently perform the search and systematic assessment of each identified study and subsequent extraction of data. Categorical random-effects meta-analyses will be conducted to obtain gender-specific pooled risk estimates. Risk of bias will be assessed using the Risk of Bias In Non-randomised Studies—of Interventions tool and the Grading of Recommendations Assessment, Development and Evaluation approach will be used to rate the quality of evidence.

**Discussion:**

This study will synthesize all available data on the gender-specific relationship between various dimensions of alcohol use and suicidal behavior simultaneously in a coherent framework. We will provide risk estimates with the detail needed to better understand the respective risk relationships and appreciate the burden of alcohol-attributable suicide.

**Systematic review registration:**

PROSPERO CRD42022320918.

**Supplementary Information:**

The online version contains supplementary material available at 10.1186/s13643-022-02159-0.

## Background

Suicide is a global public health problem, with close to 700,000 people dying by suicide every year [1]. In 2019, death by suicide accounted for more than one in every 100 deaths (1.3%) [1]. Despite international initiatives to reduce suicide mortality rates [2–4], suicide remains a leading cause of premature mortality worldwide; highlighting the need for innovative suicide prevention strategies. Alcohol use has been identified as an important risk factor for suicidal behavior [5, 6], having both precipitating and predisposing effects [7, 8]. For example, heavy alcohol consumption (> 100 g) prior to the event has been found to increase the risk of attempting suicide by up to 90 times, in comparison with abstinence [9]. Further, individuals with an alcohol use disorder (AUD) have been found to have a three-fold higher risk of both suicide attempt and death by suicide, compared to individuals without an AUD [7]. As such, interventions targeting alcohol use may be a promising area for suicide prevention. However, before this can be fully appreciated, valid risk estimates must be obtained.

Although there are a number of meta-analyses available on the risk of alcohol use on suicidal behavior [7, 8, 10–16], they have been limited to acute alcohol use and AUDs, and the vast majority have not been gender-specific to date. One exception to the latter is a recent meta-analysis by Amiri and colleagues [17] who did not find a difference by gender. However, their exposure definition did not distinguish between lifetime alcohol use or presence of AUD and thus, was inadequate. Further, a number of primary studies seem to have been missed, calling into question the validity of their meta-analysis. Thus, valid gender-specific pooled risk estimates remain to be a significant gap in the scientific literature. This is particularly important as the risk relationship between alcohol use and suicidal behavior has been shown to significantly differ by sex. For instance, women were found to have a heightened risk of death by suicide while intoxicated (a blood alcohol concentration (BAC) level of ≥ 0.08 g/dL; odds ratio = 10.0, 95% CI: 8.7–11.6), compared to men (odds ratio = 6.2, 95% CI: 5.6–6.9) [10]. Further, as members of the investigative team have shown, women with an AUD who had a treatment contact have been found to have a two-fold higher rate of death by suicide than men [15].

The finding that the association between alcohol use and suicidal behavior is more pronounced in women than in men may be due to the fact that, compared to men, women have a heightened susceptibility to the effects of alcohol [18, 19]. The heightened susceptibility is manifested by a more rapid progression, or a “telescoped course”, of AUDs [20], characterized by a shorter time from the onset of alcohol consumption to entry into treatment, and by an earlier onset of alcohol-related health and psychosocial complications [21]. Further, it has been shown that women are more likely to experience social stigmatization and weakened social integration than men for heavy drinking [22, 23]. Such social determinants could also explain women’s elevated risk of suicide, with respect to heavy alcohol use/AUDs.

Further, as specified above, meta-analyses on the predisposing effects have been largely limited to AUD [7, 14–16, 24]. However, the inclusion of average alcohol volume consumed and binge drinking will allow us to capture instances in which sub-clinical levels of drinking may also contribute to suicidal behavior risk, which may present another promising point of preventive intervention on a larger scale (e.g., through alcohol control policy), which has been neglected in the past [13, 25, 26]. Thus, this study will be the first to generate detailed and high-quality quantitative evidence syntheses on the risk of average alcohol volume consumed and binge drinking on suicidal behavior. Furthermore, we will describe and quantify the dose–response relationship between average alcohol volume consumed and suicidal behavior, filling this gap in the scientific literature.

## Objective

The objective of this systematic review and meta-analysis is to determine the gender-specific risk of suicidal behaviors (suicide attempt and death by suicide) for (1) average alcohol volume consumed, (2) binge drinking, and (3) individuals with an AUD. The following hypotheses will be tested:

1. The association between average alcohol volume and suicidal behaviors follows a dose–response relationship.
2. The risk of suicidal behavior is higher for individuals who binge drink and individuals with an AUD, compared to drinkers who do not binge drink and drinkers without an AUD, respectively.
3. The respective risk relationships are higher for women than for men.

**Fig. 1:**
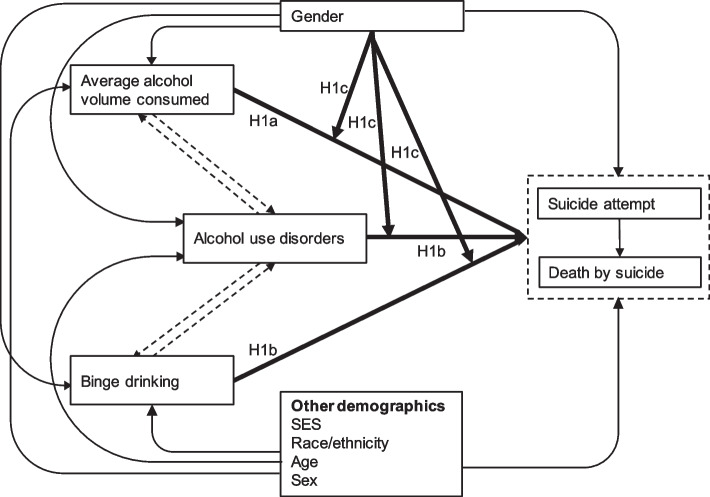
Conceptual and analytical framework

See Fig. 1 for the conceptual and analytical framework for this study.

## Methods

The current protocol is reported in accordance with the Preferred Reporting Items for Systematic Reviews and Meta-Analyses for Protocols (PRISMA-P) [27].

### Comprehensive systematic literature search

The systematic literature search and meta-analyses will be conducted and reported according to the standards set out in the most widely recognized international guidelines and protocols for such methods [28–30]. This protocol has been registered on the international prospective register of systematic reviews (PROSPERO, registration number CRD42022320918) to ensure transparency in the review process, avoid duplication of efforts, and state our objective, a priori hypotheses, and analysis plan, as described in this protocol.

#### Search strategy

A comprehensive systematic literature search will be conducted in multiple electronic databases, including Embase, PsycINFO, PubMed, and Web of Science (including Science Citation Index, Social Sciences Citation Index, and Arts and Humanities Citation Index; the search strategy in each database is provided in Additional file 1). The search will be conducted using a predetermined set of keywords (using free-text keywords and medical subject headings, wherever possible):

1. Alcohol addiction OR alcohol consum* OR alcohol use OR alcohol drinking OR alcohol intoxication OR alcohol abuse OR alcoholism OR alcohol use disorder* OR alcohol dependen* OR alcohol-related disorder* OR binge drinking OR heavy drinking OR heavy episodic drinking; AND
2. Attempted suicide OR death by suicide OR intentional self-harm OR suicid* OR suicide OR suicide attempt* OR suicide mortality.

The search will not be limited in terms of study design, year of publication, geography, or language of publication. Manual reviews of the content pages of the major epidemiological journals and citations in the articles deemed relevant, as well as the studies included in related reviews and meta-analyses, will be conducted.

#### Inclusion and exclusion criteria

Our basic inclusion criteria will be (1) an original, quantitative study; with (2) a measure of risk of at least one dimension of our alcohol exposures in relation to at least one of our outcomes of interest (suicide attempt or death by suicide), and its corresponding measure of variability is reported (or sufficient data to calculate these); and (3) estimates of risk stratified by gender. Studies (1) that use only qualitative labels of alcohol use, and (2) where suicide attempt and non-suicidal self-harm cannot be disaggregated will be excluded. See Table 1 for the specific inclusion and exclusion criteria pertaining to study characteristics.

**Table 1:** PICOS criteria for study selection

Criteria	Inclusion criteria	Exclusion criteria
Population	- Individuals ≥ 15 years of age	- None
Intervention/exposure	- Alcohol use: 1) Average alcohol volume consumed 2) Binge drinking 3) AUDs	- Qualitative labels of alcohol use, such as “social,” “moderate,” or “heavy” consumption
Comparators	1) Average alcohol volume consumed will be compared to lifetime abstention 2) Binge drinkers will be compared to drinkers who do not binge drink 3) Individuals with an AUD will be compared to drinkers without an AUD	- None
Outcomes	- Suicidal behavior: 1) Suicide attempt 2) Death by suicide - Gender-stratified risk estimates	- Deliberate self-harm is reported and suicide attempt and non-suicidal selfharm cannot be disaggregated
Study design	- Quantitative observational study designs: cohort, case–control, or cross-sectional^a^	- None
Other	- Any language, any geographical region, and any year of publication	- None

*AUD* alcohol use disorder

^a^Preference will be given to cohort studies; however, if there are not a sufficient number of cohort studies (i.e., more than five for each exposure-outcome combination), we will also include case–control studies and cross-sectional studies

We will accept studies from all settings (e.g., psychiatric and somatic in- or out-patients, colleges, general population settings, workplace settings, and others) as long as they meet the basic inclusion criteria specified above. Cohort studies have the advantage of temporal distance between exposure assessment and outcome assessment and thus have the potential to provide the strongest evidence of a causal relationship. Therefore, preference will be given to cohort studies. However, because the prevalence of suicidal behavior is rare and because we expect there to be much fewer studies that have stratified their analyses by gender, in comparison to studies that have assessed the risk for men and women combined, if there are not a sufficient number of cohort studies (i.e., more than five for each exposure-outcome combination), we will also include case–control studies and cross-sectional studies.

#### Study selection

Under the supervision of the principal investigator, two reviewers will independently perform the search and systematic assessment of each identified study and subsequent extraction of data; any disagreements will be resolved by a third reviewer of the investigative team (clinician and/or alcohol epidemiologist depending on the nature of the disagreement). At the abstract level, Population, Intervention (Exposure), Comparators, Outcomes, and Study design (PICOS) criteria outlining the characteristics of studies to be selected for further review will be applied (see Table 1). Inter-rater reliability of selection results and data extraction will be examined using Fleiss’s weighted Kappa [31]. Study selection will begin by screening titles and abstracts for inclusion. Then, full-text articles of all studies screened as potentially relevant (that is, there is an indication based on title or abstract that the article contains risk data on the association of at least one dimension of our alcohol exposures with suicide attempt or death by suicide) will be considered.

### Definitions and data sources

#### Assessment of alcohol exposure

There is a general lack of consistency in primary studies with regard to the categorization of alcohol exposure and clarification of terms is necessary to derive meaningful conclusions about the effect of alcohol on suicidal behavior [32, 33]. Accordingly, we will distinguish between average alcohol consumption, binge drinking, and AUDs in all analyses.

##### Average alcohol volume consumed

Among drinkers, we will convert reported alcohol intake categories in primary studies into an average of pure alcohol in grams per day (g/day) using the midpoints (mean or median) of reported drinking group categories. For open-ended categories, we will add ¾ of the second-highest category’s range to the lower limit of the open-ended category of alcohol intake if the mean was not reported. We will use reported conversion factors when standard drinks were the unit of measurement to convert all measures to grams (g) of pure alcohol per day [34]. In Canada, one standard drink is 13.6 g of pure alcohol, which corresponds to 12 oz (341 ml, about 5% alcohol) of beer, cider, or cooler; 5 oz (142 ml, about 12% alcohol) of wine; and 1.5 oz (43 ml, about 40% alcohol) of hard liquor (vodka, rum, whisky, gin). For our analysis, average alcohol volume consumed will be classified as follows, given data availability: (1) lifetime abstainer; (2) former drinkers; (3) occasional drinkers (less than weekly drinking or 0.1–2.49 g/day); and (4) average amount of alcohol consumed during the reference period (categorization will be based on the Canadian low-risk drinking guidelines). This categorization will make it possible to assess suicidal behavior risk with different cut-off points for men and women, reflecting Canadian low-risk drinking guidelines and differential BAC levels given the same alcohol intake. Qualitative labels, such as “social,” “moderate,” or “heavy” consumption, vary considerably between studies and lack standardization; as such, they will be excluded from quantitative analyses if the quantification of volume of alcohol intake used to define these qualitative labels is not reported. Effort will be made in all such cases to contact the corresponding author to obtain such information.

##### Binge drinking

The effect of binge drinking (i.e., irregular heavy drinking episodes, as distinguished from daily heavy drinking, which is captured by average alcohol volume consumed, as described above) will be defined as consuming four or more and five or more standard drinks during a single occasion for women and men, respectively, at least once in the past 30 days; or a BAC level of 0.08 g/dL. We will also consider exposure categories, such as usual amount of drinks per drinking day where four or more and five or more standard drinks are reported for women and men, respectively; alcohol intoxication; being drunk; having black-outs; and having heavy drinking occasions as valid assessments of binge drinking.

##### Alcohol use disorders

AUDs will be defined as per Diagnostic and Statistical Manual of Mental Disorders, third or fourth edition (alcohol abuse and alcohol dependence), and fifth edition (AUD); International Classification of Disease (ICD), version 9, 10, or 11 (harmful use of alcohol and alcohol dependence syndrome); psychiatric opinion, accepted diagnostic tools as outlined in *Assessing Alcohol Problems: A Guide for Clinicians and Researchers* [35]; and people who seek or receive AUD treatment.

#### Assessment of suicidal behavior

Suicidal behavior includes suicide attempt and death by suicide and will be defined in a manner consistent with the definitions of the Center for Disease Control and Prevention [36]. Suicide attempt will be defined as any self-inflicted injurious behavior that is intended to kill oneself but that is non-fatal, or applicable ICD-9, ICD-10, and ICD-11 codes, and death by suicide will be defined as death caused by any self-inflicted injurious behavior that was intended to kill oneself [36], or applicable ICD-9, ICD-10, and ICD-11 codes. Given that the characteristics of those who attempt suicide can be quite different from those who die by suicide, particularly with respect to gender [37], suicide attempt and death by suicide will be treated as two separate dichotomous outcomes (presence versus absence). With current definitions of deliberate self-harm being any type of self-injurious behavior, with (suicide attempt) or without (non-suicidal self-harm) the intention to kill oneself, studies where deliberate self-harm is reported and suicide attempt and non-suicidal self-harm cannot be disaggregated will be excluded.

### Data extraction

Data extraction will be piloted using ten randomly selected studies. We will extract gender-specific risk estimates of suicidal behavior in relation to the specific alcohol exposures of interest and their corresponding variances (including all estimates with varying degree of adjustment, and all reported subgroup analyses), number of cases of suicidal behavior and persons at risk or controls for each reported category of alcohol use (if not directly reported, these will be estimated based on standard formulas [38, 39]), definitions, instruments/criteria used, reference periods involved in the measurement of exposure and outcomes, study design characteristics, survey method, setting (e.g., clinical, community), sample characteristics (e.g., sex, race/ethnicity, country, age), length of follow-up, first year(s) of baseline assessment, medical and psychiatric comorbidity status, and adjustment for any other covariates.

### Risk of bias and quality appraisal

We will conduct a risk of bias analysis by rating primary studies as having low, moderate, and high risk of bias using the Risk of Bias In Non-randomised Studies—of Interventions (ROBINS-I) tool [40]. Grading of Recommendations Assessment, Development, and Evaluation (GRADE) will be used to evaluate the quality of evidence [41], and standard “Summary of Findings” tables will be generated using the GRADEpro software [42]. The body of evidence will begin with a high-certainty rating; however, the body of evidence will be downgraded by two levels due to the inherent risk of bias associated with the lack of randomization, as recommended by Schünemann and colleagues [43]. In addition to risk of bias, consideration of lowering certainty further will involve the assessment of inconsistency, indirectness, imprecision, and publication bias. The following domains will subsequently be used to upgrade the certainty level of the body of evidence: large effects, dose–effect relations, and when plausible residual confounders or other biases increase certainty. Additionally, for each dichotomous meta-analytic estimate derived, we will estimate 95% prediction intervals, which evaluates the uncertainty of the effect that would be expected in a new study addressing the same association [44, 45]. Evidence of excess of significant findings will be assessed by the Ioannidis test [46]. All discrepancies in quality ratings will be reconciled by the principal investigator. Inter-rater reliability of quality appraisals will also be examined using Fleiss’s weighted Kappa [31].

### Data management

All articles identified in the database searches will be exported into EndNote 20 [47] and duplicate articles will be identified and removed. The remaining articles will then be exported into Covidence [48] for screening and full-text review.

### Statistical analysis

To obtain gender-specific pooled risk ratio (RR) estimates, categorical random-effects meta-analyses [49] will be conducted with hazard ratios, relative risks, standardized mortality ratios, and odds ratios treated as equivalent measures of risk. Given that the characteristics of those who attempt suicide can be quite different from those who die by suicide, particularly with respect to gender [37], suicide attempts and death by suicide will be treated as two separate binary outcomes in all analyses. If necessary, effect sizes within studies will be re-calculated to contrast alcohol exposure categories against a common reference group for each aim [50]. Between-study heterogeneity will be assessed using Cochran’s Q [51] and the *I*^2^ statistic [52]. If identified, potential sources of heterogeneity (e.g., exposure definition, adjustment for confounders such as psychiatric disorders [mood disorders, other substance use disorders], age, diagnostic criteria, education, population, race/ethnicity, social environment [living alone, social isolation, and marital status], study design) will be examined. This will be done in a narrative review or by comparing different adjustments in meta-regression models, depending on data availability. Publication bias will be examined by visually inspecting the funnel plot and Egger’s weighted regression-based test [53]. A sensitivity analysis will be conducted using the leave-one-out approach, where we will iteratively remove one study at a time to confirm that our findings are not being driven by any single study. In case evidence for publication bias is detected, we will use the trim-and-fill method to estimate an adjusted effect size [54]. All meta-analyses will be conducted on the natural log scale in Stata, version 16 [55], and α = 0.05 (two-tailed) will be considered statistically significant. To evaluate the robustness of the effect sizes and the plausibility of a causal relationship, we will conduct *E*-value analyses [56, 57]. The *E*-value approach evaluates the plausibility of uncontrolled confounding by quantifying the effect size that is required to account for the observed effects. We will analyze the effect sizes that unmeasured confounds would need to have with both the exposure and the outcome to fully explain the association. A high *E*-value suggests that an association is at least partially causal.

For *hypothesis 1*, mid-points (means) of reported exposure categories will be used to assign exposure values when only category boundaries were reported. We will use restricted cubic spline regression in two-stage multivariate random-effects inverse variance-weighted meta-regression models to derive the best-fitting dose–response curve estimates, and 95% CI [58–62]. To investigate the role of binge drinking and AUD (*hypothesis 2*), the binge drinking group and AUD group will be contrasted against non-binge drinkers and people without an AUD, respectively. In the case where a study does not present risk estimates for AUDs overall, but rather for separate AUD diagnoses (e.g., one for alcohol abuse and one for alcohol dependence), we will first pool them in a fixed effects meta-analysis and include the resulting risk estimate in the main meta-analysis. To test *hypothesis 3*, categorical random-effects meta-analyses [49] will be conducted to obtain gender-specific pooled RR estimates, and the given chi-square test statistic and associated probability that the pooled RR ratio (women to men) is equal to one will be used.

### Incorporation of sex and gender

As indicated throughout, gender is an important factor to consider as a potential moderator when examining the effects of alcohol use on suicidal behavior, given that the prevalence of both varies considerably between men and women, as do the risk relationships. As such, all analyses will be gender-specific (i.e., men and women, as well gender-diverse individuals, if possible). We are aware that the relationships of interest can also vary by sex, as the biological effects of alcohol may be related to risk of suicidal behavior. Although we suspect that in most cases studies have captured gender only, second-level disaggregation by sex will be done to explore the role of sex beyond gender, if possible.

## Discussion

This study will provide the gender-disaggregated estimates of the risk relationship between various dimensions of alcohol use and suicidal behavior, which are urgently needed to inform program design and implementation. Without such data, the understanding of the relationship between alcohol use and suicidal behavior remains incomplete, as currently available knowledge syntheses often mask sub-population disparities–i.e., risk disparities across genders. Ultimately, with the collection and estimation of gender-disaggregated risk estimates, we will be able to subsequently model the impact of scalable individual-level interventions targeting alcohol use as indirect suicide preventive strategies. Accordingly, stakeholders and policymakers will be better equipped to strategically plan and implement suicide prevention programs to improve outcomes with respect to alcohol misuse.

Even though alcohol use and suicidal behavior are linked, alcohol use is an often-overlooked risk factor for suicidal behavior; in fact, alcohol use-related suicide risk has been called an unmet public health crisis [63] and a missed opportunity [64] in suicide prevention. Furthermore, the need for more gender-specific research on the association between alcohol use and suicidal behavior has been highlighted [65]. Thus, this study is timely, as it is designed to fill an identified gap in the scientific literature and will provide findings with high clinical and public health importance.

## Supplementary Information

**Additional file 1. **Search strategy in each electronic database.

## Data Availability

Not applicable.

## Abbreviations

AUD: Alcohol use disorder
BAC: Blood alcohol concentration
RR: Risk ratio
