# Supplementary material for: Alcohol use and the gender-specific risk of suicidal behavior: a systematic review and meta-analysis protocol
Source: Syst Rev. 2022 Dec 23;11:279. doi: 10.1186/s13643-022-02159-0 (PMC9783973; doi:10.1186/s13643-022-02159-0)
Supplement: Supplementary file 1 — Additional file 1. Search strategy in each electronic database. [file 13643_2022_2159_MOESM1_ESM.docx]

**Additional file 1**

**Search strategy in each electronic database**

**Embase**

(exp alcohol abstinence/ or exp alcohol consumption/ or exp alcohol blood level/ or exp alcohol intoxication/ or exp "Alcohol Use Disorders Identification Test"/ or exp alcohol abuse/ or (Alcohol drink* or alcohol intoxication or alcohol abuse or alcohol dependen* or alcohol-related disorder* or heavy episodic drink* or "alcohol use" or alcohol addiction or alcohol consum* or binge drink* or heavy drink* or alcoholi* or alcohol misuse or alcohol abstinen* or alcohol intake or blood alcohol or drunkenness*).ab,kw,ti.) and (exp suicide attempt/ or exp suicide/ or (suicid* or self-harm or self-injur*).ab,kw,ti.)

**PsycINFO**

(exp Blood Alcohol Concentration/ or exp Alcoholism/ or exp Alcohol Drinking Patterns/ or exp "alcohol use disorder"/ or exp Alcohol Drinking Attitudes/ or (blood alcohol content or alcoholic intoxication or alcoholism or drinking behavior).mh. or (Alcohol drink* or alcohol intoxication or alcohol abuse or alcohol dependen* or alcohol-related disorder* or heavy episodic drink* or "alcohol use" or alcohol addiction or alcohol consum* or binge drink* or heavy drink* or alcoholi* or alcohol misuse or alcohol abstinen* or alcohol intake or blood alcohol or drunkenness*).ab,id,ti.) and (exp Suicide/ or suicide.mh. or (self harm or suicid* or self-injur*).ab,id,ti.)

**PubMed**

("suicide"[MeSH Terms] OR ("self-harm"[Title/Abstract] OR "suicid*"[Title/Abstract] OR "self injur*"[Title/Abstract])) AND ("alcohol drink*"[Title/Abstract] OR "alcohol intoxication"[Title/Abstract] OR "alcohol abuse"[Title/Abstract] OR "alcohol dependen*"[Title/Abstract] OR "alcohol related disorder*"[Title/Abstract] OR "heavy episodic drink*"[Title/Abstract] OR "alcohol use"[Title/Abstract] OR "alcohol addiction"[Title/Abstract] OR "alcohol consum*"[Title/Abstract] OR "binge drink*"[Title/Abstract] OR "heavy drink*"[Title/Abstract] OR "alcoholi*"[Title/Abstract] OR "alcohol misuse"[Title/Abstract] OR "alcohol abstinen*"[Title/Abstract] OR "alcohol intake"[Title/Abstract] OR "blood alcohol"[Title/Abstract] OR "drunkenness*"[Title/Abstract] OR "blood alcohol content"[MeSH Terms] OR "alcoholic intoxication"[MeSH Terms] OR "alcoholism"[MeSH Terms] OR "drinking behavior"[MeSH Terms])

**Web of Science**

(TS=("Alcohol drink*" OR "alcohol intoxication" OR "alcohol abuse" OR "alcohol dependen*" OR "alcohol-related disorder*" OR "heavy episodic drink*" OR "alcohol use" OR "alcohol addiction" OR "alcohol consum*" OR "binge drink*" OR "heavy drink*" OR "alcoholi*" OR "alcohol misuse" OR "alcohol abstinen*" OR "alcohol intake" OR "blood alcohol" OR "plasma alcohol" OR "drunkenness*")) AND (TS=(suicid* OR self-harm* OR self-injur*))
